# *Lactobacillus paragasseri* OLL2809 Improves Premenstrual Psychological Symptoms in Healthy Women: A Randomized, Double-Blind, Placebo-Controlled Study

**DOI:** 10.3390/nu15234985

**Published:** 2023-12-01

**Authors:** Asako Sato, Akika Fukawa-Nagira, Toshihiro Sashihara

**Affiliations:** Food Microbiology and Function Research Laboratories, Division of Research and Development, Meiji Co., Ltd., Hachioji 192-0919, Tokyo, Japan; akika.fukawa@meiji.com (A.F.-N.); toshihiro.sashihara@meiji.com (T.S.)

**Keywords:** *Lactobacillus paragasseri* OLL2809, probiotics, premenstrual symptoms, MDQ, arousal, irritability, activity, hypothalamic-pituitary-adrenal axis

## Abstract

*Lactobacillus paragasseri* OLL2809 has been shown to ameliorate stress. This study employed a randomized, placebo-controlled, double-blind, parallel-group design to assess the efficacy of continuous ingestion of OLL2809 for managing menstrual symptoms in healthy women. Eighty healthy adult women aged 25–40 years who experienced premenstrual and menstrual symptoms were randomly assigned to either the OLL2809 or placebo group (*n* = 40 each) and ingested tablets containing OLL2809 or placebo for three menstrual cycles. The OLL2809 group exhibited a significantly greater change in premenstrual ‘arousal’ scores on the menstrual distress questionnaire compared to the placebo group after the three menstrual cycles. Specifically, changes in the ‘activity’ subfactor were significantly higher in the OLL2809 group than in the placebo group. Additionally, the OLL2809 group reported significantly lower premenstrual irritability on the visual analog scale than the placebo group. These results suggest that OLL2809 may contribute to enhancing the quality of life of women.

## 1. Introduction

Menstruation is a natural physiological process that occurs in women of reproductive age. However, menstrual-related symptoms, which result from short-term fluctuations or unstable levels of female hormones, present significant health challenges for women [1,2,3,4,5,6]. Recent meta-analyses indicate that premenstrual symptoms are highly prevalent, affecting approximately half of the women worldwide [7]. In this meta-analysis, the prevalence of premenstrual symptoms was the lowest (12%) in France and the highest (98%) in Iran [2,8]. Dysmenorrhea was the most common symptom, with a prevalence of 85%, followed by psychological complaints, and tiredness. In a Dutch survey, 38% of women reported that they were unable to perform all their regular daily activities during their menstrual period [3]. In Australia, 44% of women have moderate or severe dysmenorrhea, which affects their work performance and causes absenteeism [6]. In the case of Japan, an internet survey involving approximately 20,000 Japanese women, aged 15–49 years, who menstruate, found that 74% experienced menstrual-related symptoms [9]. The estimated annual economic burden of menstrual symptoms in Japan is approximately 683 billion Japanese yen (approximately 8.6 billion US dollars), with a reported productivity loss of 72% [9]. Hence, menstrual-related symptoms are a critical concern not only for women but also for society.

Despite extensive research, many uncertainties remain in understanding the cause of premenstrual syndrome (PMS), one of the common menstrual-related symptoms. Currently, no known relationship exists between PMS and organic abnormalities or abnormalities in ovarian hormone secretion. Consequently, there are currently no clinically applicable biomarkers for diagnosis [10]. While some individuals find relief through self-care practices, treatment becomes necessary when self-care falls short. Treatment options include counseling, lifestyle guidance, exercise therapy, and pharmacotherapy. Pharmacological treatments for symptom relief include diuretics, sedatives, herbal medicines, selective serotonin reuptake inhibitors (SSRIs), and oral contraceptives [11]. However, in Japan, only 20% of women seek medical care for menstruation-related issues, with a large proportion opting for over-the-counter pain relief solutions [9]. If dietary choices can help alleviate menstrual symptoms, it would be a valuable means to enhance the quality of life (QOL) of women.

*Lactobacillus paragasseri* OLL2809 (formerly reported as *Lactobacillus gasseri*; hereafter referred to as OLL2809) is a probiotic with immunomodulatory effects [12]. Clinical trials conducted on patients with endometriosis demonstrated its effectiveness in reducing menstrual pain [13]. Moreover, clinical trials involving university student athletes have demonstrated its efficacy in reducing anxiety and stress [14]. Animal experiments have also confirmed that OLL2809 administration can alter the gut microbiota and ameliorate stress-induced depression-like behaviors [15]. Conversely, it is widely acknowledged that stress can affect female reproductive function through various pathways, with stress reported as a major causative factor for menstrual-related symptoms [16,17,18]. Additionally, recent evidence has shed light on the role of gut microbiota in PMS [19]. Hence, OLL2809 has the potential to alleviate menstrual-related symptoms, particularly psychological ones. This study aimed to evaluate the effects of OLL2809 on menstrual-related symptoms in healthy women.

## 2. Materials and Methods

### 2.1. Study Design

We conducted a randomized, double-blind, placebo-controlled, parallel-group trial at a single site, the Fukuda Clinic in Osaka, Japan, with clinical support from Soiken Inc. (Osaka, Japan), a clinical research organization, between June 2021 and September 2022. The study complied with the ethical principles outlined in the Declaration of Helsinki. This study was approved by the Institutional Review Board of Fukuda Clinic (Review Number: IRB-20210619-3) and the Meiji Institutional Review Board (Review Number: 202). The study protocol was registered in the University Hospital Medical Information Network Clinical Trials Registry (ID: UMIN000044933), and all participants provided written informed consent.

### 2.2. Participants

This study enrolled healthy women who met the following criteria: (1) aged 25–40 years, (2) had a menstrual cycle of 25–38 days with a menstrual period lasting 3–7 days, and (3) experienced menstrual-related symptoms before and during menstruation. Participants with menstrual-related symptoms were defined in this study as having a total score of 1 or higher on the menstrual distress questionnaire (MDQ) [20] both before and during menstruation at primary screening. Exclusion criteria included: (1) pregnancy, lactation, or planning to become pregnant during the study; (2) a history of gynecologic disorders or having gynecologic disorders at the time of the study (such as hypermenorrhea, secondary amenorrhea, dysmenorrhea, endometriosis, uterine fibroids, PMS, premenstrual dysphoric disorder (PMDD), breast cancer, cervical cancer, uterine body cancer, and ovarian cancer); (3) food allergy; (4) diarrhea due to the ingestion of dairy products; (5) the use of drugs (including Chinese herbal medicines or oral contraceptive pills), supplements, or foods containing lactic acid bacteria, such as yogurt, for more than four days a week; (6) habitual consumption of analgesics prophylactically before the onset of menstrual pain; (7) severe menstrual pain that cannot be alleviated with commercially available analgesics; (8) classification as a psychosomatic disease (type IV) in the Japanese edition of the Cornell Medical Index Health Questionnaire (J-CMI) [21]; (9) a stress score of 20 or more on the Stress Checklist KM (SCL-KM) [22]; (10) the absence of menstrual-related symptoms; and (11) disqualification by the physician for other reasons.

The effect size of OLL2809 in this study was predicted to be 0.7 based on a previous study involving patients with endometriosis [13]. With a significance level of 5% and power of 80%, the required sample size was calculated to be 66; the number of participants per group was 33 for a total target sample size of 80 participants. This accounted for potential exclusions from the efficacy analysis and an estimated dropout rate of approximately 10%.

The primary screening included blood and urine sampling, anthropometric measurements, a lifestyle questionnaire, J-CMI, SCL-KM, MDQ, and medical interviews with participants who provided consent. From the participants meeting the inclusion criteria and not meeting the exclusion criteria, 120 participants with higher MDQ total scores (combined physical and psychological symptom scores) in the primary screening were selected. Furthermore, as the secondary screening, 80 participants with MDQ total scores close to the median at baseline were selected. Menstrual symptoms are known to vary significantly among individuals [23]. This two-step selection procedure was to enroll participants with similar severity in the symptoms.

### 2.3. Intervention

The intervention consisted of ingesting tablets containing OLL2809 (referred to as active tablets) or a placebo. The active tablets were composed of dried OLL2809 powder manufactured at our plant (100 mg per two tablets, equivalent to approximately 1 × 10^10^ bacterial cells), maltitol, cellulose, calcium carboxymethyl cellulose, calcium stearate, and silicon dioxide. The placebo tablets contained lyophilized OLL2809 culture medium powder and dextrin instead of OLL2809 powder. These tablets were prepared by API Co., Ltd. (Gifu, Japan). The Institutional Review Board confirmed that the placebo and active tablets could not be distinguished based on their appearance, odor, or taste. The participants were instructed to ingest two tablets once daily from day 5 of their menstruation cycle 0 to day 4 of their menstruation cycle in cycle 3, which spanned approximately 12 weeks. The intervention was performed for three menstrual cycles, in line with a previous study that found the clinical efficacy of this strain after 12 weeks of ingestion [13].

### 2.4. Questionnaires

#### 2.4.1. MDQ

We evaluated the efficacy of OLL2809 on premenstrual and menstrual symptoms by using the MDQ, a 46-item self-administered questionnaire designed to assess and treat premenstrual and menstrual symptoms. This questionnaire was developed by Moos in 1968 [20], has been extensively validated for its reliability, and remains the most commonly used self-report instrument for measuring menstrual cycle symptoms [24]. The validity of the Japanese version was confirmed by Akiyama et al. in 1979 [25]. The 46 items were categorized into eight subfactors: (1) pain, (2) water retention, (3) autonomic reactions, (4) negative affect, (5) concentration, (6) behavioral change, (7) arousal, and (8) control. Notably, only subfactor (7) “arousal” captured positive changes. The participants rated the severity of each symptom as follows: ‘none’ (0 points), ‘slight’ (1 point), ‘moderate’ (2 points), ‘strong’ (3 points), or ‘very strong’ (4 points). Individual scores for each subfactor were summed to calculate the total subfactor score. Scores for (1) pain, (2) water retention, (3) autonomic reactions, and (8) control were summed to obtain the physical symptom score, while scores for (4) negative affect, (5) concentration, and (6) behavioral change were summed, and (7) arousal was subtracted to derive the psychological symptom score.

#### 2.4.2. Medical Outcomes Study 36-Item Short-Form Health Survey Version 2 (SF-36)

The SF-36 acute version [26] was used to assess health-related QOL. The responses were processed using the web-based scoring program of Qualitest Co., Ltd. (Kyoto, Japan), which categorized and scored them as physical component summary (PCS), mental component summary (MCS), and role/social component summary (RCS). Higher values indicate better health.

#### 2.4.3. Visual Analog Scale (VAS)

Between seven days before the expected date of menstruation and four days after menstruation, the participants recorded the degrees of five symptoms once daily: (1) lower abdominal cramps, (2) skin disorders, (3) swelling, (4) irritability, and (5) mood swings on a 100 mm scale from 0 (no symptoms) to 100 (severe symptoms experienced). The maximum values during the seven days from before menstruation to the day before menstruation were designated as ‘premenstrual’ values, while the maximum values during the four days from the first day to the fourth day of menstruation were designated as ‘menstrual’ values. However, if the premenstrual survey period was less than three days due to variations in the predicted date, it was considered missing data.

#### 2.4.4. Analgesic Use Scores

Analgesic use scores were calculated based on the diary records. These scores were assigned as follows: 0 points for no use of analgesics, 1 point for one use, 2 points for two uses, and 3 points for three uses or more. Values for the seven days from seven days before menstruation to the day before menstruation were categorized as ‘premenstrual’ values, while values for the four days from the first day to the fourth day of menstruation were categorized as ‘menstrual’ values.

### 2.5. Study Protocol

**Figure 1:**
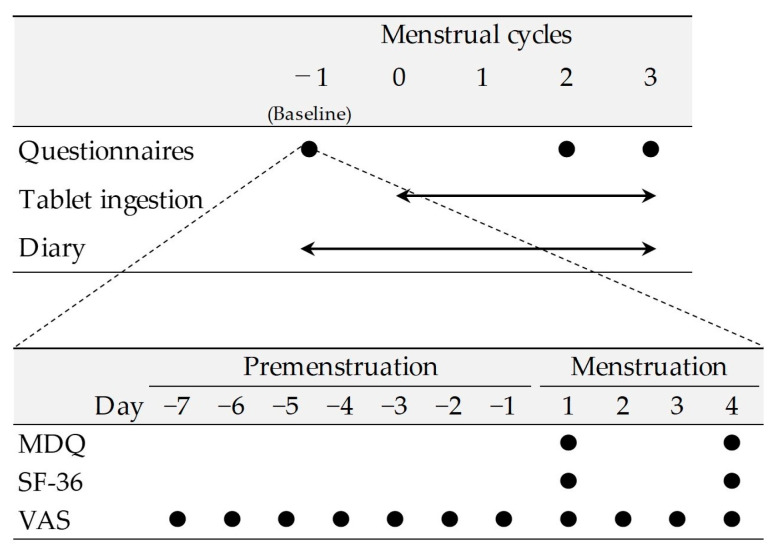
Study schedule.

This study spanned five menstrual cycles (cycle −1 to 3) and followed the schedule outlined in Figure 1. MDQ and SF-36 were administered on the first and fourth days of menstruation. The participants were instructed to report their subjective physical and psychological symptoms from seven days before the onset of menstruation to the day before menstruation on the first day of menstruation. The participants also recorded their perceived physical and psychological symptoms from the first day of menstruation until the fourth day of menstruation, on the fourth day of menstruation. VAS assessments were performed from seven days before the expected date of the menstrual cycle −1 to the fourth day of menstruation. The MDQ, SF-36, and VAS assessments were conducted during menstruation in menstrual cycles 2 and 3, as well as in menstrual cycle −1. Throughout the study period, the participants were asked to maintain a life diary documenting their ingestion of the test food, subjective symptoms of body condition changes, treatment received, medications used, and health foods used.

Throughout the study period, the participants were instructed to avoid extreme dietary changes involving excessive dieting or overeating that substantially deviated from their usual eating habits. Additionally, the participants were instructed to not use analgesics for prophylactic purposes. To differentiate between temporary menstrual symptoms in healthy women and persistent discomfort caused by gynecological conditions, the research investigator emphasized the importance of promptly seeking medical advice if premenstrual or menstrual distress significantly interfered with daily activities, or if these symptoms persisted not only before or during menstruation, but also after menstruation.

### 2.6. Randomization and Blinding

The allocation supervisor (Statcom Co., Ltd., Tokyo, Japan), who had no direct involvement in the study, organized five teams of participants based on their menstrual cycle timing to ensure that the intervention was timed to fit each participant’s menstrual cycle. They were then randomly assigned into two groups using a computer-generated random sequence for each team. It was also confirmed that there were no differences in the participants’ age and the MDQ total score during the premenstrual and menstrual periods of menstrual cycle −1 between the assigned groups. This study was conducted in a double-blind manner, with test foods and measurement data concealed from both the research investigators and the participants throughout the study.

### 2.7. Outcome

The primary outcome of the study focuses on changes in the MDQ score from baseline at menstrual cycle 3, whereas the secondary outcomes includes alterations in the SF-36 and VAS scores.

### 2.8. Safety Assessment

Safety assessments involved monitoring the number of adverse events and reactions. In this study, adverse events were defined as newly occurring unfavorable or unintended injuries or signs of illness that emerged from the initiation of test food ingestion until the conclusion of the study.

### 2.9. Statistical Analysis

The data are presented as the mean and standard deviation (SD) or standard error (SE). All analyses were conducted using a two-tailed test, with a significance level of 5%. The participant baseline data were compared between groups using Student’s *t*-test or the Mann–Whitney U-test for continuous data and Fisher’s exact test or chi-squared test for categorical data.

For the efficacy assessment, multiple regression analysis was performed with changes from menstrual cycles −1 to 3 as the dependent variable. The explanatory variables included the group (placebo group = 0, OLL2809 group = 1), the analgesic use score for menstrual cycle −1, the stress score, and regular exercise habits (at least once a week; absent = 0, present = 1). These variables were chosen because of the significant differences in analgesic use scores between the two groups in this study. Previous research has indicated that stress and exercise habits can influence menstrual symptoms [18,27]. All explanatory variables had a variance expansion factor (VIF) of 10 or less, ensuring that there were no issues related to multiple collinearities between the explanatory variables. The adjusted estimates of between-group differences are presented with 95% confidence intervals (CIs). In other analyses, Student’s *t*-tests were used for group comparisons, and paired *t*-tests were employed for pre- and post-intervention comparisons. According to the pre-specified protocol, all analyses were conducted using the per-protocol set (PPS). An analysis of the primary outcome was also conducted for the full analysis set (FAS) population. Adverse events were evaluated using the FAS. All statistical analyses were performed using SPSS version 26 (IBM Corp., Armonk, NY, USA).

## 3. Results

### 3.1. Participant Selection

**Figure 2:**
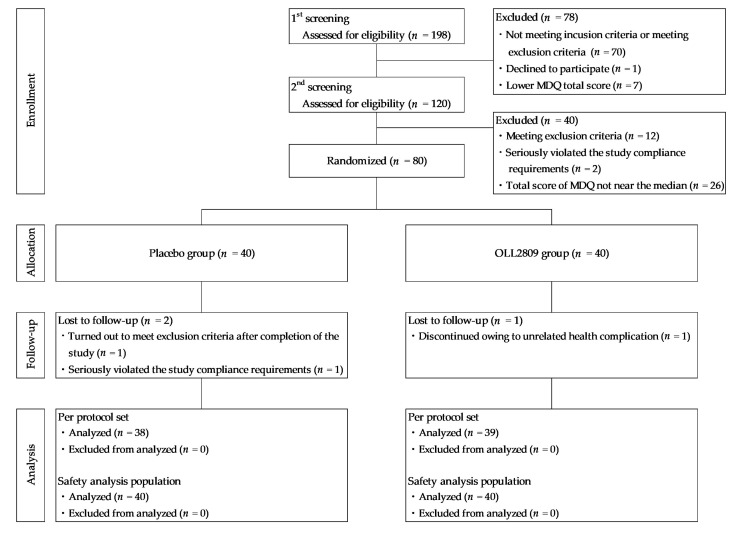
Flow chart of participants in this study.

Figure 2 illustrates a flowchart detailing the selection process for the study participants. The primary screening involved 198 participants (age (year): 32.6 ± 4.2 (mean ± SD), 25–39 (min-max); premenstrual MDQ total scores: 15.7 ± 15.1, 0–87; menstrual MDQ total scores: 19.3 ± 16.3, 0–95), with 120 participants (age: 32.3 ± 4.3, 25–39; premenstrual MDQ total scores: 16.5 ± 13.9, 1–87; menstrual MDQ total scores: 19.5 ± 16.3, 3–94) proceeding to secondary screening. Of these, 80 participants (age 32.4 ± 4.5, 25–39; premenstrual MDQ total scores 12.6 ± 10.8, 1–87; menstrual MDQ total scores 14.3 ± 9.5, 3–63) were divided into two groups, each consisting of 40 participants. Two individuals in the placebo group were withdrawn from the study: one due to the emergence of exclusion criteria after study completion, and the other due to a serious breach of compliance requirements. One participant in the OLL2809 group withdrew due to an unrelated health complication. Consequently, there were 38 participants in the placebo group and 39 in the OLL2809 group for the PPS analysis. As no participants were excluded from the FAS analysis other than those who withdrew from the study, the number of endpoints was the same for the FAS and PPS analyses, and the statistical analysis results were also the same. The results of the PPS analysis are presented below.

Baseline characteristics between the placebo and OLL2809 groups, including age; body mass index; age at menarche; menstrual cycle; menstrual period; alcohol consumption; stress score; MDQ score before; during, and after menstruation; J-CMI; number of smokers; regular exercise habits; and premenstrual analgesic use score, were not significantly different (Table 1). However, the analgesic use score during menstruation was significantly higher in the placebo group than in the OLL2809 group (*p* = 0.018). The mean percentage of test food ingestion was 98.3% in the placebo group and 97.8% in the OLL2809 group, with no significant differences between the groups.

### 3.2. Effectiveness of OLL2809 on Premenstrual Symptoms

**Figure 3:**
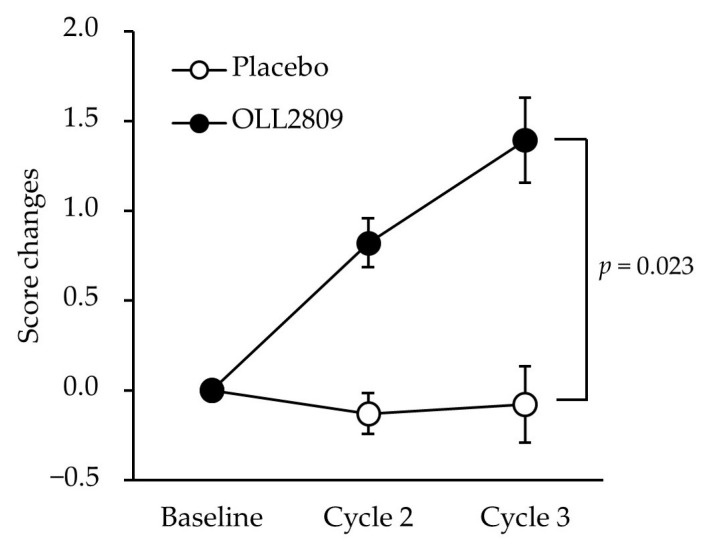
Time-course change in premenstrual ‘arousal’ score, a subfactor in MDQ, during the study period. Score changes adjusted for the group, analgesic use score, stress score, and regular exercise habit. Error bars indicate 95% CI of the mean predicted values.

**Figure 4:**
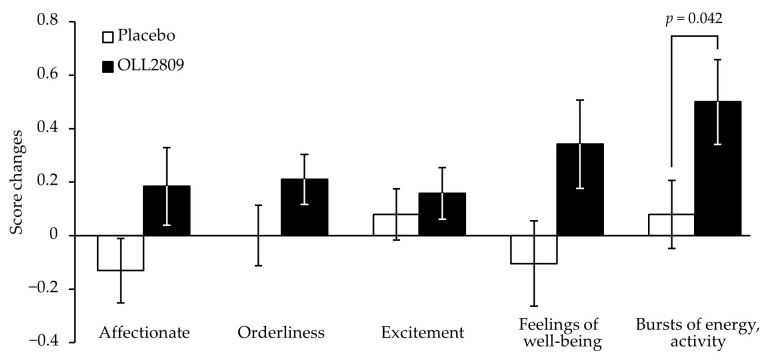
Change in specific items of premenstrual ‘arousal’ score, a subfactor in MDQ, at menstrual cycle 3 from the baseline. Each data point represents the mean value with SE (*n* = 38 in the placebo group and *n* = 38 in the OLL2809 group).

Table 2 shows the changes in MDQ scores during the premenstrual period and the treatment differences estimated using multiple regression analysis in the PPS analysis. The baseline values of the FAS are shown in Table S2. No significant differences were observed in physical or psychological scores between the two groups. However, OLL2809 ingestion had a significant positive effect on the score changes in the ‘arousal’ subfactor, values minus baseline (Table 2; *p* = 0.023). The score changes were consistently higher in the OLL2809 group than in the placebo group throughout the study period, and the difference became statistically significant in cycle 3 (Figure 3; *p* = 0.023). Further analysis of the ‘arousal’ subfactor revealed that the item ‘bursts of energy, activity’ was significantly higher in the OLL2809 group compared to the placebo group (Figure 4; *p* = 0.042). There were no significant differences between the groups in the items of ‘affectionate’, ‘orderliness’, ‘excitement’, and ‘feelings of well-being’.

**Figure 5:**
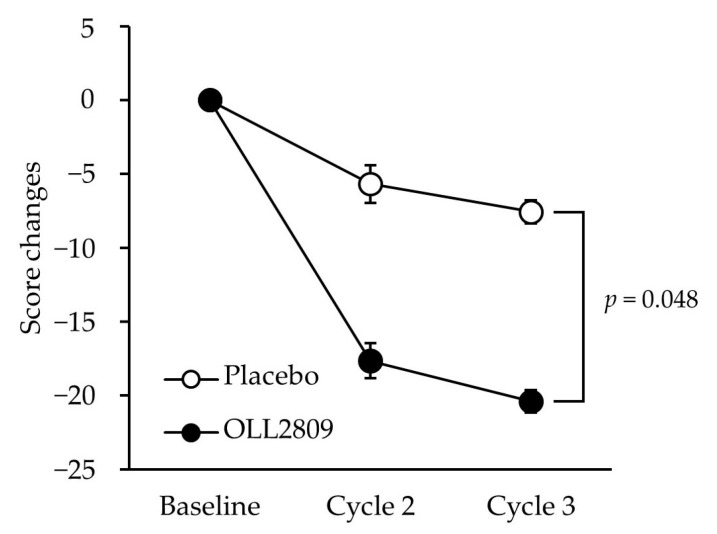
Time-course change in VAS of premenstrual irritability score during the study period. Score changes adjusted for group, analgesic use score, stress score, and regular exercise habit. Error bars indicate 95% CI of the mean predicted values.

Table 3 shows the SF-36 and VAS scores during the premenstrual period. No significant differences were found in SF-36 scores between the groups. In contrast, the changes in the VAS scores for ‘irritability’ in the OLL2809 group during the three menstrual cycles were significantly greater than those in the placebo group (Figure 5; *p* = 0.048). The changes in ‘mood swings’ after three menstrual cycles tended to be greater in the OLL2809 group than in the placebo group (*p* = 0.070). No significant differences were observed between the groups in terms of physical symptoms, such as ‘lower abdominal cramps’, ‘skin disorders’, and ‘swelling’.

### 3.3. Effectiveness of OLL2809 on Menstrual Symptoms

Table S3 shows the MDQ scores during menstruation in the PPS analysis. The baseline values of the FAS are shown in Table S4. No significant differences were observed in the physical or psychological scores between the two groups. Similarly, there were no significant differences in the MDQ subfactors between the groups.

The SF-36 and VAS scores during menstruation are shown in Table S5. During menstruation, there were no notable differences in any of the evaluation parameters between the groups.

### 3.4. Safety

No serious adverse events were observed during this study. Newly identified adverse events during the intervention period were 34 in 13 participants in the placebo group and 34 in 16 participants in the OLL2809 group. There were no significant differences in the numbers between the groups. Adverse events included fever, chills, joint pain, runny nose, nasal congestion, sneezing, cough, sore throat, hoarse voice, phlegm, nausea, abdominal pain, diarrhea, vomiting, anorexia, stomach discomfort, fatigue, lethargy, malaise, dizziness, insomnia, headache, back pain, ear pain, skin irritation, rough hands, arm pain, leg pain, gum pain and swelling, swelling of the legs, and stye. All adverse events were determined to be unrelated to the ingestion of the test food because their causes were evident and considered transient or accidental by the physician in charge.

## 4. Discussion

We conducted a randomized, placebo-controlled, double-blind, parallel-group study involving healthy women experiencing subjective menstrual symptoms, with the test food being OLL2809. Analgesics are the most frequently used treatment for menstrual pain in Japan. Consequently, this study was meticulously structured to minimize the effect of analgesic use on the evaluation of menstrual symptoms. This was achieved by excluding individuals with a tendency to proactively take analgesics before the onset of menstrual pain and instructing participants not to use analgesics prophylactically before symptom onset. However, participants were not restricted from using analgesics when symptoms appeared. As a results, the use of analgesics was anticipated to be a significant confounding variable, and there were notable differences in the analgesic use scores between the groups at baseline (Table 1). For statistical analysis, a multiple regression approach was employed, incorporating the analgesic use score as an explanatory variable, given its influence on various other menstrual-related symptoms. In terms of our primary endpoint, the OLL2809 group exhibited a significantly higher ‘arousal’ score in the premenstrual MDQ compared to the placebo group following the three-cycle intervention. The substantial improvement in premenstrual activity in the OLL2809 group was particularly noteworthy. In the secondary outcome, as assessed using the VAS for ‘irritability’, the OLL2809 group displayed a significant decrease in scores before menstruation in comparison to the placebo group. These findings provide compelling evidence that OLL2809 ingestion has a beneficial effect on menstrual-related symptoms. Furthermore, it is imperative to acknowledge that the study participants were healthy adult women. As such, we deliberately excluded individuals diagnosed with specific conditions, such as PMS, PMDD, dysmenorrhea, or endometriosis. Additionally, those who were deemed to be experiencing symptoms of sufficient severity to disrupt their daily lives were excluded because of the possibility of an underlying medical condition, as previously explained. Consequently, the results of this study hold significant meaning in the context that they offer an alternative beyond pharmaceuticals for “women who experience psychological symptoms before menstruation but may not necessarily require medical treatment”.

Although the pathogeneses of PMS and PMDD remain unclear, they have been reported to be linked to cyclic variations in estrogen and progesterone levels, accompanied by a decrease in serotonin secretion [28,29]. The pulsatile secretion of gonadotropin-releasing hormone (GnRH) is crucial for the secretory cycle of estrogen and progesterone. Hypothalamic GnRH secretion is known to be suppressed by the corticotropin-releasing factor, which is secreted in response to stress [30]. Stress has been identified as a major factor influencing PMS development [17,18]. The multiple regression analyses in this study confirmed that the stress scores at baseline were significant influencing factors among the premenstrual MDQ subfactors, including (1) pain; (3) autonomic reactions; (4) negative affect; and (7) arousal. Furthermore, OLL2809 has demonstrated the potential to reduce stress. The administration of OLL2809 increased beneficial microbes, such as *Bifidobacterium*, *Lactobacillus*, and *Akkermansia*, ameliorated depression-like behaviors in stressed mice, and induced neurite outgrowth in the hippocampal dentate gyrus [15]. The brain–gut–microbiota axis has gained significant attention in recent years because of its impact on PMS and the psychological manifestations associated with PMDD. Probiotics may play a therapeutic role in managing premenstrual disorders by restoring the intestinal ecosystem, modulating sex hormones, regulating the renin–angiotensin–aldosterone system, secreting serotonin and gamma-aminobutyric acid, and preventing systemic inflammation [19]. Minelli et al. investigated the effects of *Lactobacillus acidophilus* and *Bifidobacterium bifidum* ingestion for two months on gastrointestinal and psychological symptoms in young women with PMS [31]. They reported that these probiotics contributed to maintaining the activity of sex hormone-metabolizing enzymes in the gut and the homeostasis of the gut microbiota, which may modulate PMS manifestations, leading to the alleviation of symptoms. Similarly, Nishida et al. reported that *Lactobacillus gasseri* CP2305 ingestion improved premenstrual psychological symptoms in young women [32]. They suggested the potential for estrogenic variability through alterations in the gut microbiota due to CP2305 ingestion as a mechanism of action in addition to controlling stress responsiveness through the HPA axis. Objective biomarkers for the efficacy of menstrual symptoms were not evaluated in this study, as widely accepted clinical diagnoses are lacking. However, the efficacy of OLL2809 in ameliorating menstrual symptoms may involve mechanisms related to the regulation of stress responses through the HPA axis.

In this study, the improvement effect of OLL2809 was primarily observed on psychological symptoms before menstruation, whereas there was a limited effect on physical symptoms before and during menstruation. The relationship between the physical and psychological symptoms associated with menstruation has been widely reported. Symptoms such as headache and migraine, resulting from neurovascular disorders due to estrogen withdrawal before menstruation, and lower abdominal pain, associated with uterine contractions induced by prostaglandins can affect psychological symptoms such as mood swings and irritation [33]. Reports evaluating the association between physical symptoms of the menstrual cycle and premenstrual depressive symptoms have shown strong linear and moderate curvilinear effects when physical symptom scores were used as predictors of depressive symptoms. Physical and depressive symptoms were not associated with lower levels of physical symptoms; however, this association was stronger at higher levels of physical symptoms [29]. In our study, the VAS score for ‘lower abdominal cramps’ in all participants at baseline was significantly higher during menstruation (46.1 ± 2.8) compared to the premenstrual period (19.2 ± 2.3) (*n* = 77, *p* < 0.001). The ameliorative effect of OLL2809 on menstrual symptoms appears to be more pronounced during the premenstrual period when the degree of physical symptoms is relatively low and may not have as significant an impact on physical and psychological symptoms influenced by them during menstruation when the symptoms are more severe than those before menstruation.

The first limitation was the possibility of selection bias owing to the selection of participants with moderate MDQ total scores. Although external validity may be compromised, we believe that the study population is not extremely biased; therefore, it is possible to observe this effect in the study population. Secondly, the MDQ, SF-36, and VAS scores used as endpoints were subjective assessments. Given the absence of objective biomarkers for menstrual symptoms, the mechanism of action of OLL2809 remains unclear based on the current results. We speculate that this lack of clarity may be related to the stress response of the HPA axis. However, further investigation is necessary to elucidate how OLL2809 ingestion improves premenstrual psychological symptoms.

## 5. Conclusions

This study suggests that the consumption of OLL2809 over three menstrual cycles in healthy women can alleviate premenstrual ‘decline in activity’ and ‘irritability’, thereby indicating the potential of OLL2809 to enhance women’s QOL.

## Figures and Tables

**Table 1:** Baseline characteristics of the participants (per-protocol set).

Characteristics	Placebo Group (*n* = 38)	OLL2809 Group (*n* = 39)	*p*-Value
Age (year)	32.4 ± 4.6	32.4 ± 4.4	0.972 ^a^
Body mass index (kg/m^2^)	21.3 ± 3.3	20.1 ± 2.6	0.085 ^a^
Age at menarche (year)	12.4 ± 1.3	12.8 ± 1.7	0.235 ^a^
Menstrual cycle (day)	29.7 ± 2.5	29.7 ± 1.8	0.863 ^a^
Menstrual period (day)	5.6 ± 1.2	5.5 ± 0.9	0.701 ^a^
Alcohol consumption (g/day)	13.0 ± 14.5	14.4 ± 13.1	0.653 ^a^
Stress score	2.8 ± 2.6	2.8 ± 2.9	0.995 ^a^
MDQ total score			
Premenstruation	12.7 ± 13.7	12.4 ± 7.1	0.913 ^a^
During menstruation	14.7 ± 11.9	14.2 ± 6.9	0.822 ^a^
After menstruation	2.2 ± 3.6	2.0 ± 2.4	0.791 ^a^
J-CMI (%)			
I	33 (86.8)	30 (76.9)	0.217 ^b^
II	4 (10.5)	9 (23.1)	
III	1 (2.6)	0 (0.0)	
Smoker (%)	2 (5.3)	0 (0.0)	0.240 ^c^
Regular exercise habits (%)	9 (23.7)	13 (33.3)	0.349 ^b^
Analgesic use score			
Premenstruation	0.1 ± 0.3	0.1 ± 0.2	0.625 ^d^
During menstruation	0.7 ± 1.0	0.3 ± 0.9	0.018 ^d^

Data are shown as mean ± SD or number (percentage). ^a^ Student’s *t*-test. ^b^ Chi-squared test. ^c^ Fisher’s exact test. ^d^ Mann–Whitney U test. Abbreviations: MDQ, menstrual distress questionnaire; J-CMI, the Japanese edition of the Cornell Medical Index Health Questionnaire.

**Table 2:** Mean scores and changes during the study period, and estimated treatment differences for MDQ in the premenstrual period (per-protocol set).

Factors	Group	Baseline	Cycle 2	Cycle 3	Score Changes (Cycle 3—Baseline)	Estimated Treatment Difference		
						β [95% CI]		*p*-Value
Physical score	Placebo	9.9 ± 0.8	6.8 ± 0.8 ^†^	7.4 ± 0.7 ^†^	−2.5 ± 0.7	0.33	[−2.44–3.09]	0.815
	OLL2809	10.3 ± 1.0	7.4 ± 0.7 ^†^	7.5 ± 0.9 ^†^	−2.6 ± 1.2			
Psychological score	Placebo	8.6 ± 1.4	5.2 ± 1.4 ^†^	5.3 ± 1.4 ^†^	−3.3 ± 1.4	−0.34	[−5.29–4.60]	0.891
	OLL2809	8.3 ± 1.3	7.3 ± 1.8	4.6 ± 1.7	−3.6 ± 2.0			
Subfactors								
Pain	Placebo	4.8 ± 0.5	3.4 ± 0.4 ^†^	3.8 ± 0.6	−1.1 ± 0.6	−0.25	[−1.94–1.44]	0.767
	OLL2809	5.0 ± 0.5	3.5 ± 0.3 ^†^	3.5 ± 0.4 ^†^	−1.6 ± 0.6			
Water retention	Placebo	4.1 ± 0.4	2.8 ± 0.4 ^†^	2.9 ± 0.4 ^†^	−1.1 ± 0.4	−0.12	[−1.41–1.18]	0.857
	OLL2809	4.2 ± 0.5	3.2 ± 0.3 ^†^	2.8 ± 0.3 ^†^	−1.3 ± 0.5			
Autonomic reactions	Placebo	0.8 ± 0.2	0.5 ± 0.2	0.5 ± 0.1	−0.3 ± 0.1	0.33	[−0.27–0.93]	0.275
	OLL2809	0.8 ± 0.2	0.5 ± 0.2	0.7 ± 0.2	−0.1 ± 0.3			
Negative affect	Placebo	4.6 ± 0.8	3.7 ± 0.9	3.4 ± 0.8	−1.1 ± 0.7	−0.86	[−3.18–1.47]	0.467
	OLL2809	5.2 ± 0.7	4.5 ± 0.9	3.3 ± 0.6 ^†^	−1.8 ± 0.9			
Concentration	Placebo	3.4 ± 0.6	1.8 ± 0.4 ^†^	2.0 ± 0.4 ^†^	−1.4 ± 0.5	1.32	[−0.49–3.12]	0.149
	OLL2809	2.8 ± 0.4	3.3 ± 0.6	2.6 ± 0.6	−0.2 ± 0.7			
Behavioral change	Placebo	2.4 ± 0.4	1.3 ± 0.4 ^†^	1.5 ± 0.4 ^†^	−0.8 ± 0.4	0.58	[−0.65–1.81]	0.350
	OLL2809	2.0 ± 0.3	2.1 ± 0.4	1.8 ± 0.4	−0.2 ± 0.4			
Arousal (positive)	Placebo	1.7 ± 0.4	1.6 ± 0.4	1.6 ± 0.5	−0.1 ± 0.4	1.38	[0.20–2.57]	0.023 *
	OLL2809	1.7 ± 0.4	2.5 ± 0.5 ^†^	3.1 ± 0.6 ^†^	1.4 ± 0.5			
Control	Placebo	0.3 ± 0.1	0.1 ± 0.0	0.2 ± 0.1	−0.1 ± 0.1	0.37	[−0.14–0.88]	0.157
	OLL2809	0.3 ± 0.1	0.2 ± 0.1	0.5 ± 0.2	0.3 ± 0.2			

Baseline (placebo, *n* = 38; OLL2809, *n* = 39), cycle 2 (placebo, *n* = 38; OLL2809, *n* = 39), cycle 3 (placebo, *n* = 38; OLL2809, *n* = 38). Data are shown as mean ± SE. The estimates were driven by multiple regression analysis. β: standardized partial regression coefficient. ^†^ *p* < 0.05 vs. Baseline by paired *t*-test. * *p* < 0.05, multiple regression analysis.

**Table 3:** Mean scores and changes during the study period, and estimated treatment differences in SF-36 and VAS in the premenstrual period (per-protocol set).

Factors	Group	Baseline	Cycle 2	Cycle 3	Score Changes (Cycle 3—Baseline)	Estimated Treatment Difference		
						β [95% CI]		*p*-Value
SF-36								
PCS	Placebo	51.8 ± 1.6	52.6 ± 1.3	52.5 ± 1.5	0.7 ± 1.5	−0.29	[−4.08–3.51]	0.881
	OLL2809	53.9 ± 1.0	53.9 ± 0.9	53.9 ± 1.0	0.0 ± 1.2			
MCS	Placebo	51.3 ± 1.4	53.7 ± 1.2	54.5 ± 1.2 ^†^	3.2 ± 1.3	0.77	[−2.90–4.43]	0.679
	OLL2809	50.8 ± 1.0	52.3 ± 1.4	54.5 ± 1.3 ^†^	3.8 ± 1.2			
RCS	Placebo	49.6 ± 1.5	51.7 ± 1.5	50.3 ± 1.8	0.7 ± 1.9	−0.16	[−5.44–5.12]	0.951
	OLL2809	48.0 ± 1.3	49.8 ± 1.3	49.2 ± 1.3	1.2 ± 1.8			
VAS								
Lower abdominal cramps	Placebo	16.9 ± 3.2	16.8 ± 3.2	10.2 ± 2.6	−7.0 ± 3.9	−1.15	[−11.27–8.97]	0.821
	OLL2809	21.3 ± 3.4	15.5 ± 3.1	11.4 ± 2.6 ^†^	−9.0 ± 2.8			
Skin disorders	Placebo	25.8 ± 3.1	21.0 ± 3.5	17.2 ± 2.8 ^†^	−8.7 ± 3.0	−6.42	[−16.43–3.59]	0.205
	OLL2809	35.6 ± 3.8	23.1 ± 2.9 ^†^	17.6 ± 2.5 ^†^	−17.4 ± 3.9			
Swelling	Placebo	27.4 ± 4.3	18.1 ± 3.4 ^†^	18.3 ± 2.9 ^†^	−9.5 ± 4.3	0.84	[−9.64–11.31]	0.874
	OLL2809	26.9 ± 3.7	18.2 ± 3.3 ^†^	17.3 ± 3.3 ^†^	−9.6 ± 2.7			
Irritability	Placebo	27.2 ± 3.7	20.8 ± 4.4	19.1 ± 4.0	−7.6 ± 4.9	−13.63	[−27.12–−0.14]	0.048 *
	OLL2809	40.9 ± 4.4	25.7 ± 3.6 ^†^	20.7 ± 3.6 ^†^	−20.2 ± 4.2			
Mood swings	Placebo	19.5 ± 3.7	16.5 ± 4.0	18.0 ± 4.3	−1.1 ± 4.9	−12.93	[−26.95–1.09]	0.070
	OLL2809	29.0 ± 4.2	20.1 ± 3.8 ^†^	14.2 ± 3.4 ^†^	−14.2 ± 4.6			

Baseline (SF-36: placebo, *n* = 38; OLL2809, *n* = 39; VAS: placebo, *n* = 37; OLL2809, *n* = 38), cycle 2 (SF-36: placebo, *n* = 38; OLL2809, *n* = 39; VAS: placebo, *n* = 37; OLL2809, *n* = 37), cycle 3 (SF-36: placebo, *n* = 38; OLL2809, *n* = 39; VAS: placebo, *n* = 38; OLL2809, *n* = 38). Data are shown as mean ± SE. The estimates were driven by multiple regression analysis. β: standardized partial regression coefficient. ^†^ *p* < 0.05 vs. Baseline by paired *t*-test. * *p* < 0.05, multiple regression analysis. Abbreviations: PCS, physical component summary; MCS, mental component summary; RCS, role/social component summary.

## Data Availability

Data supporting the results of this study are available from the corresponding author upon reasonable request.

## Supplementary Materials

The following supporting information can be downloaded from https://www.mdpi.com/article/10.3390/nu15234985/s1. Table S1: CONSORT 2010 checklist of information to include when reporting a randomized trial. Reference [34] is cited in the Table S1. Table S2: Mean MDQ scores during the premenstrual period (full analysis set). Table S3: Mean scores and changes during the study period, and estimated treatment differences for MDQ in the menstrual period (per-protocol set). Table S4: Mean MDQ scores during the menstrual period (full analysis set). Table S5: Mean scores and changes during the study period, and estimated treatment differences for SF-36 and VAS in the menstrual period (per-protocol set).
